# The Effect of High Glucocorticoid Administration and Food Restriction on Rodent Skeletal Muscle Mitochondrial Function and Protein Metabolism

**DOI:** 10.1371/journal.pone.0005283

**Published:** 2009-04-20

**Authors:** Y. Nancy You, Kevin R. Short, Marion Jourdan, Katherine A. Klaus, Stephane Walrand, K. Sreekumaran Nair

**Affiliations:** Endocrine Research Unit, Mayo Clinic College of Medicine, Rochester, Minnesota, United States of America; University of Alabama, United States of America

## Abstract

**Background:**

Glucocorticoids levels are high in catabolic conditions but it is unclear how much of the catabolic effects are due to negative energy balance versus glucocorticoids and whether there are distinct effects on metabolism and functions of specific muscle proteins.

**Methodology/Principal Findings:**

We determined whether 14 days of high dose methylprednisolone (MPred, 4 mg/kg/d) Vs food restriction (FR, food intake matched to MPred) in rats had different effects on muscle mitochondrial function and protein fractional synthesis rates (FSR). Lower weight loss (15%) occurred in FR than in MPred (30%) rats, while a 15% increase occurred saline-treated Controls. The *per cent* muscle loss was significantly greater for MPred than FR. Mitochondrial protein FSR in MPred rats was lower in soleus (51 and 43%, respectively) and plantaris (25 and 55%) than in FR, while similar decline in protein FSR of the mixed, sarcoplasmic, and myosin heavy chain occurred. Mitochondrial enzymatic activity and ATP production were unchanged in soleus while in plantaris cytochrome c oxidase activity was lower in FR than Control, and ATP production rate with pyruvate + malate in MPred plantaris was 28% lower in MPred. Branched-chain amino acid catabolic enzyme activities were higher in both FR and MPred rats indicating enhanced amino acid oxidation capacity.

**Conclusion/Significance:**

MPred and FR had little impact on mitochondrial function but reduction in muscle protein synthesis occurred in MPred that could be explained on the basis of reduced food intake. A greater decline in proteolysis may explain lesser muscle loss in FR than in MPred rats.

## Introduction

Mitochondria are the major intracellular site for fuel oxidation and ATP production, and undergo adaptive changes in response to a variety of energy demands. Changes in mitochondrial function may impact protein metabolism since protein synthesis and ubiquitin-dependent protein degradation are ATP-dependent. Catabolic conditions are associated with negative energy balance and increased levels of catabolic hormones, including glucocorticoids. Such catabolic conditions are associated with energy deficit and accelerated protein loss. However, the impact of glucocorticoids and energy restriction on muscle mitochondrial function and protein metabolism is incompletely defined.

Several prior reports demonstrated that high doses of glucocorticoids can produce significant muscle wasting within 3–11 days in rats [1]–[7]. A greater effect is typically observed in white, predominantly glycolytic muscles, when compared to red, oxidative muscles (e.g. soleus) [5]–[9]. Although muscle protein synthesis rate was reduced in these glucocorticoid-treated animal models [2], [3], [5], [8], [10], little or no deficit in mitochondrial gene expression or activity of oxidative enzymes has been observed [4], [6]. This suggests that mitochondria may be less responsive to the effects of glucocorticoids and thus, one purpose of the present study was to determine whether synthesis rate of mitochondrial proteins varies from other muscle protein fractions in glucocorticoid-treated rats.

However, high-dose glucocorticoids are known to induce anorexia when administered to rodents [8], [11] so it is unclear how much of the glucocorticoid-induced changes in muscle protein synthesis are related to reduced food intake. Short-term fasting or energy restriction is known to suppress muscle protein synthesis rates in both rodents and humans [1], [2], [12], [13]. Available evidence suggests that the mitochondrial fraction may be relatively preserved during energy restriction since there was no change in mitochondrial oxidative capacity following 24 h fasting in mice [14], [15], 7 days [16], or 7 months [17] of moderate food restriction in rats, or chronic energy restriction in yeast [18]. There are reports, however, showing that activity of mitochondrial enzymes and gene transcripts may actually increase in rat muscle during 3 weeks of moderate energy restriction [16]. Nevertheless, these findings point to potentially differential regulation of muscle mitochondrial proteins during the catabolic conditions of glucocorticoid treatment or food restriction.

We tested the hypothesis that glucocorticoid treatment and food restriction have a selective effect on synthesis and function of specific muscle protein units, particularly in the mitochondria. We also sought to determine whether some or all of the effects of glucocorticoids are related to reduced food intake and whether treatment effects vary between predominantly oxidative (i.e. soleus) and mixed-composition (i.e. plantaris) skeletal muscles in the rat.

## Methods

### Animals

Male Sprague-Dawley rats (Harlan Laboratories, Indianapolis, IN) that were approximately 8 weeks old and weighing 260–300 grams initially were used for these studies. Study protocol and procedures were approved by the Institutional Animal Care and Use Committee and followed the guidelines of the National Research Council's Guide for the Care and Use of Laboratory Animals. Rats were housed individually in plastic boxes with standard bedding under a 12 h: 12 h light: dark cycle. Water and standard commercial rat chow (Purina AIN-93G, 15% total energy from protein, 25% from fat, 60% from carbohydrate) were provided. Food intake and body mass were monitored daily throughout the study.

### Study protocol

Rats were randomly assigned to one of the three study groups of eight animals each: 1) Saline-treated controls (Control), 2) Food restricted (FR), or 3) Methylprednisolone-treated (MPred). On protocol *Day 1* each animal was anesthetized with pentobarbital (Nembutal, 40 mg/kg; Abbot Laboratories, Chicago, IL) via intra-peritoneal injection, and a mini-osmotic pump (Aztec 2002, Alza Scientific Products, Palo Alto, CA) was subcutaneously implanted in the dorsal neck region. Pumps implanted in Control and FR rats delivered sterile normal saline at 12 µl/day (as specified by the pump model) for 14 days. The MPred group received methylprednisolone (Solu-Medrol 40 mg Act-O-Vial System, Pharmacia & Upjohn, Peapeck, NJ) at 4 mg/kg/day for 14 days. Control and MPred rats had *ad libitum* access to food. MPred rats decreased food intake from baseline, as shown in earlier studies [1], [2], [7], [8] and in order to achieve comparable food intake, the FR group was supplied with the same amount of food as consumed by the MPred animals (pair-feeding). Animals in the FR group began the experiment approximately 1 week after the corresponding animals in the MPred group to allow for pairfeeding. The amount of food provided to individual FR animals was matched to a corresponding animal in the MPred group for each experimental day. Body weight and food consumption were recorded each day in the middle of the 12 hour light cycle. Food was kept in tip-resistant bowls. Bowl weight and any visible food on the cage bottom was used to calculate food intake. In this study we did not determine whether the temporal pattern of food intake varied among groups.

On the morning of protocol *Day 14*, food was removed 3 h before infusion studies began. To measure protein synthesis rate [*ring*-^13^C_6_] phenylalanine (Cambridge Isotope Laboratories, Cambridge MA, 99 atom percent excess, 15 mg/kg) was infused via the tail vein. At 20 min individual hindlimb skeletal muscles (soleus, plantaris, quadriceps) were rapidly removed under pentobarbital anesthesia. Samples were blotted of blood and visible fat and connective tissue was removed. A portion of soleus and plantaris muscles were kept on ice in saline-soaked gauze for mitochondrial studies and the remainder was quickly frozen in liquid nitrogen and stored at −80 C. Blood was collected via cardiac puncture, separated into serum (for insulin concentration) or plasma (for glucose and amino acids) and frozen until further analysis.

Because it was critical to immediately excise a portion of the fresh muscle for mitochondrial function analyses and then rapidly freeze the remaining tissue for protein analyses, it was not possible to measure the wet muscle weight in the initial experiments. We therefore replicated the study with another set of 24 rats (8 Control, 8 FR and 8 MPred) under identical study conditions to obtain data on total wet muscle weight immediately following tissue collection.

### Blood analysis

Serum glucose was measured using a glucose oxidase method (Beckman Instruments, Chaska, MN). Insulin was measured with a two-site immunoenzymatic method performed on the Access automated immunoassay system (Beckman Instruments, Chaska, MN).

### Mitochondrial function

Mitochondria were isolated concurrently from the soleus and plantaris by differential centrifugation as previously described [19]. Briefly, samples were homogenized in buffer containing 100 mM KCl, 50 mM Tris, 5 mM MgCl2, 1.8 mM ATP and 1 mM EDTA and spun at 720×g at 4 C. The, supernatant was spun at 10,000×g and the resulting pellet was washed and spun at 9,000×g. The final pellet was suspended in 180 mM sucrose, 35 mM KH_2_PO_4_, 10 Mg acetate, 5 mM EDTA and kept on ice. Mitochondrial ATP production rate (MAPR) was measured with a bioluminescent assay [19]–[21]. Mitochondria were added to cuvettes containing ATP-monitoring reagent (AMR, formula SL; BioThema AB, Haninge, Sweden), oxidizable substrates, and ADP. Substrates added (in mM final concentration) were: a) 1 pyruvate plus 1 malate, or b) 0.05 palmitoyl-L-carnitine plus 1 malate. All MAPR reactions were analyzed in duplicate at 25 C in a BioOrbit 1251 luminometer (BioOrbit Oy, Turku, Finland). Internal calibration was achieved by the addition of an ATP standard. MAPR was calculated after measuring the activity of citrate synthase in the mitochondrial sample and the whole tissue [6], [19], [20]. Activity of citrate synthase (CS) and cytochrome c oxidase (COX) was measured using spectraphotometric assays [20], [22].

### Muscle protein synthesis rate

Mitochondrial, sarcoplasmic and myosin heavy chain fractions were separated from both soleus and plantaris muscle samples (100–150 mg each) of each animal, as previously described [23]–[26]. Briefly, mitochondrial and sarcoplasmic protein fractions were isolated using a similar homogenization and centrifugation approach as used for the MAPR assay. Myosin heavy chain was purified using a continuous elution, polyacrylamide gel electrophoresis method. Separate 25 mg pieces of the same muscles were used to isolate the total mixed protein and tissue-free fluid fractions [25]–[27]. Total protein concentration was measured with a colorimetric assay (DC Protein Assay, BioRad Laboratories, Hercules, CA). Protein samples were hydrolyzed for 24 h in 0.05 mol/L HCl at 110 C in the presence of cation-exchange resin (AG 50W-X8, BioRad Laboratories, Hercules, CA) [28]. Amino acids from the protein hydrolysates and tissue fluid fractions were then purified over a column of the same resin. Samples were derivatized using N-methyl-N(t-butyldimethylsilyl)-trifluoroacetamide in acetonitrile. Isotopic enrichment in protein-bound amino acids was measured using gas chromatography-combustion-isotope ratio mass spectrometry (Finigan-MAT, Bremen Germany) [29]. Enrichment of free amino acids in tissue fluid and plasma was measured using gas chromatography-mass spectrometry [25], [29]. Plasma analysis was limited to 7 Control, 6 MPred and 7 FR animals due to sample availability.

Fractional protein synthesis was calculated as:
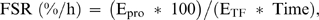
where E_pro_ and E_TF_ are the isotopic enrichments in muscle protein and in tissue fluid respectively, and Time is tracer incorporation in hours.

### Activity of branched-chain amino acid aminotransferase (BCAAT) and α-keto acid dehydrogenase (BCKAD)

Activity of BCAAT and BCKAD was measured as an index of branched-chain amino acid oxidation, which has been shown to be increased during energy restriction [30]. Since soleus and plantaris muscle samples of adequate mass were no longer available from the primary group of animals, the deep (red) portion of the quadriceps femoris were used, as previously described [31], [32]. Briefly, muscles were homogenized in a buffer, pH 7.4, containing 20 mM EDTA, 20 mM EGTA, 0.4% CHAPSO, 5 mM dithiothreitol (DTT), 25 mM HEPES and protease inhibitor cocktail (Complete Mini, Roche Applied Science, Indianapolis, IN). After two freeze-thaw cycles, samples were centrifuged at 15,000 *g* for 10 min at 4 C. The supernatant was used to measure BCAAT and BCKAD by monitoring the change in NADH absorbance at 37 C. The BCAAT reaction mixture contained 5 µM pyridoxal 5′-phosphate, 5 mM ammonium sulfate, 0.05 mM NADH, 5 mM dithiothreitol, 5 mM α-ketoglutarate, 10 mM L-leucine, 0.5 mM guanosine 5′-triphosphate, and 50 µg leucine dehydrogenase (LeuDH) in 100 mM potassium phosphate buffer. Due to the presence of ammonia, NADH and α-ketoglutarate in the BCAAT mixture, endogenous glutamate dehydrogenase (GDH) activity may interfere with the measurement of BCAAT activity. Therefore, BCAAT activity was estimated as the difference between the absorbance change in reaction with (measure of GDH plus BCAAT activity) and without (measure of GDH activity only) LeuDH included [32]. The BCKAD reaction mixture contained 5 µM pyridoxal 5′-phosphate, 5 mM ammonium sulfate, 0.05 mM NADH, 5 mM dithiothreitol and 4.5 mM α-ketoisocaproate in 100 mM potassium phosphate buffer.

### Statistics

The number of animals per group was chosen based on data for mitochondrial protein synthesis rate and ATP production from a prior study [20], [33]. Power to detect differences in other outcomes may be lower. Differences among treatment groups for most of the data were analyzed using one-way analysis of variance. Repeated measures analysis of variance was used for food intake and body mass. Where appropriate, pairwise comparisons among means were performed using Tukey's post-hoc test. In all cases, P-values less than 0.05 were considered statistically significant.

## Results

### Food intake and body mass (Fig. 1)

**Figure 1:**
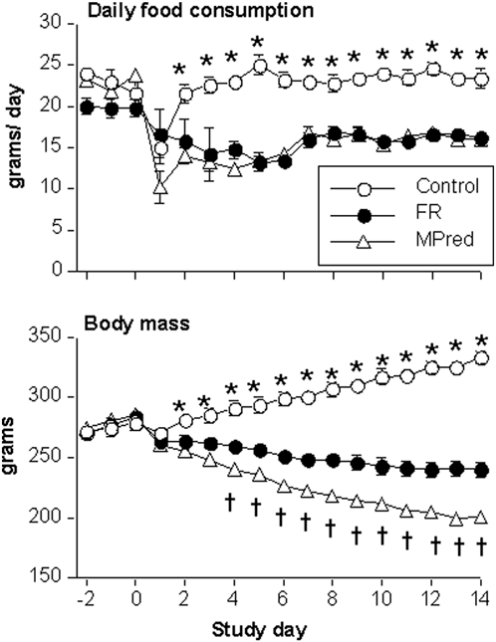
Daily food consumption and body mass (mean±SEM). FR, food restricted group; MPred, methylprednisolone group. * Control higher than MPred and FR, *P<0.05*, † MPred less than FR, *P<0.05*.

Food consumption prior to pump implantation was similar among the groups at 20–25 g/day and declined on the day after pump implantation. Food intake in the Control group recovered within 2 days and remained within 5% of the baseline value through *Day 14*. In contrast, the MPred group consumed 31% less food versus Control (*P<0.001*) during the entire treatment period. By design, food intake of FR animals matched that of the MPred animals. FR animals ate all of the food provided.

Initial body mass did not differ among the groups (Control = 272±5 g, MPred = 274±4 g, FR = 271±5 g, P>0.88). By *Day 14* body mass of Control animals increased 15% from baseline while FR rats decreased 15%, and MPred rats decreased 30%. In pairwise comparisons there were significant differences in final body mass among each of the three groups (*P<0.001*).

### Glucose and insulin concentration

Plasma glucose concentration at the end of the study in Control animals was 10.3±0.5 mmol/l and serum insulin concentration was 244±44 pmol/l. In comparison to the Control group, FR animals had significantly reduced insulin (43±7 pmol/l, *P<0.01*) and tended to have lower glucose (8.3±0.8 mmol/l, *P* = *0.070*). In MPred rats, both glucose (18.1±1.6 mmol/l) and insulin (453±33 pmol/l) concentrations were elevated (*P<0.01*) compared to the Control and FR groups.

### Muscle mass (Table 1)

As noted above, all measurements except muscle mass were acquired from the same set of animals. Muscle mass was measured in a repeat experiment conducted under identical conditions. Compared to Control animals, soleus muscle mass in the FR group was not significantly different, while plantaris was 15% lower (P<0.03). A larger difference was evident in the MPred group, as soleus was 19% lower than Control and plantaris was 48% lower than Control (both p<0.01). Additionally, both of these muscles were smaller for the MPred group compared to those of the FR group (P<0.01). A portion of each muscle was weighed before and after freeze-drying. No differences were observed for final dry weight or percent water content (Control = 75.4±1.0%, MPred = 75.9±0.7 and FR = 76.2±1.1). These data demonstrate that lower muscle mass in the MPred group was not due to dehydration secondary to prednisolone-induced hyperglycemia.

**Table 1:** Muscle weight, mitochondrial enzyme activity, and ATP production rate

	Control	FR	MPred
**Soleus**			
Weight (mg)	137.6±6.4	126.6±2.8	111.3±3.9*#
CS	34.45±1.32	32.29±1.39	33.02±1.05
COX	15.96±2.33	13.89±0.71	14.96±0.43
MAPR (PM)	9.66±0.26	10.44±0.41	10.33±0.50
MAPR (PCM)	8.00±0.32	8.61±0.32	8.94±0.76
**Plantaris**			
Weight (mg)	383. 6±19.6	326.5±8.1*	199.1±7.2*#
CS	15.41±0.46	13.68±0.73	13.31±0.89
COX	11.09±1.10	7.47±0.53 *	8.64±1.47
MAPR (PM)	7.13±0.21	7.07±0.66	5.12±0.82 *
MAPR (PCM)	3.00±0.16	3.20±0.25	2.86±0.51

FR, food restricted; MPred, methylprednisolone; CS, citrate synthase activity; COX, cytochrome c oxidase activity; MAPR, mitochondrial ATP production rate; PM, pyruvate + malate; PCM, palmitoyl-L-carnitine + malate. Activity values given as µmol/min/g tissue. Similar treatment effects for enzymes and MAPR were evident when the data were expressed relative to protein content (not shown).

* Less than Control, (*P<0.05*);

# Less than FR (P<0.05).

### Mitochondrial function

In the soleus there were no differences among treatment groups for the activity of CS or COX enzymes, or for MAPR (Table 1). In the plantaris, COX activity was 32% lower in the FR group compared to Control (*P<0.05*). MAPR with pyruvate + malate was 28% lower in the MPred plantaris compared to Control but there were no differences among groups in plantaris MAPR with palmitoyl-L-carnitine + malate.

### Muscle protein metabolism

The concentration of total mixed muscle proteins in the Control soleus was 180±4 µg/mg. There was a non-significant trend for lower values in FR (169±4 µg/mg, *P* = *0.08* versus Control) and MPred (169±5 µg/mg, *P* = *0.10* versus Control). In the plantaris, protein concentration was 201±4 µg/mg tissue in Controls, 190±4 µg/mg in FR (*P* = *0.07* versus Control) and 189±3 µg/mg in MPred (*P* = *0.03* versus Control).

Tracer enrichment in proteins and muscle tissue fluid are shown in Table 2. With one exception (soleus mixed protein) the protein-bound tracer enrichment was significantly lower in the FR and MPred groups compared to Control for each tissue fraction (total mixed, mitochondria, sarcoplasmic, and MHC) in both soleus and plantaris. Protein-bound enrichment was also significantly lower in the MPred versus FR for all soleus protein fractions and for total and mitochondrial fractions in plantaris. Tissue fluid [^13^C_6_] phenylalanine enrichment did not differ between the Control and MPred groups, but was significantly higher in both FR soleus and plantaris versus Control and MPred muscles. Consistent with previous studies [27], [34] plasma [^13^C_6_] phenylalanine enrichment was lower (p<0.05) than muscle tissue fluid in all groups, but the differences among groups for plasma [^13^C_6_] phenylalanine (Control, 9.75±1.33 molar per cent excess; FR, 18.17±1.82; MPred, 9.45±1.21) was the same as for tissue fluid.

**Table 2:** Enrichment of [^13^C_6_] phenylalanine in plasma, muscle proteins and tissue fluid

	Control	FR	MPred
**Plasma**	9.75±1.33	18.17±1.82*	9.45±1.21†
**Soleus**			
Total mixed protein	0.0383±0.0038	0.0332±0.0018	0.0211±0.0014 * †
Mitochondrial protein	0.0857±0.0043	0.0668±0.0069 *	0.0423±0.0027 * †
Sarcoplasmic protein	0.0499±0.0018	0.0393±0.0034 *	0.0272±0.0012 * †
Myosin heavy chain	0.0365±0.0025	0.0207±0.0034 *	0.0117±0.0010 * †
Tissue fluid	11.74±0.74	18.56±1.76 *	13.00±1.19 †
**Plantaris**			
Total mixed protein	0.0359±0.0010	0.0207±0.0011 *	0.0157±0.0017 * †
Mitochondrial protein	0.0539±0.0033	0.0381±0.0027 *	0.0321±0.0020* †
Sarcoplasmic protein	0.0293±0.0014	0.0160±0.0012 *	0.0146±0.0010 *
Myosin heavy chain	0.0252±0.0036	0.0118±0.0013 *	0.0094±0.0038 *
Tissue fluid	13.27±0.71	23.54±1.36*	11.36±0.71 †

Enrichment values for plasma and tissue fluid as molar percent excess, for proteins given as atom percent excess. FR, food restricted; MPred, methylprednisolone.

* Different from Control, *P<0.05*;

† Less than FR, *P<0.05.*

**Figure 2:**
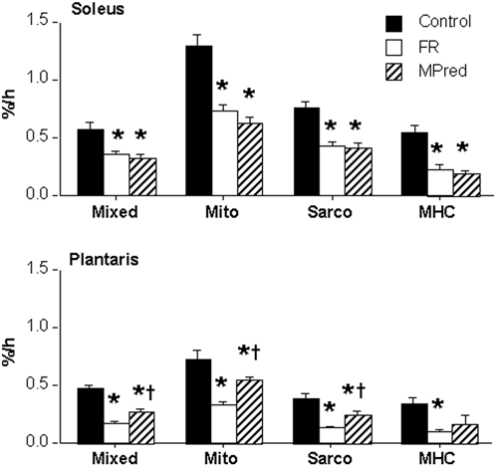
Fractional synthesis rates (FSR) of muscle proteins in soleus and plantaris. Data shown as mean±SEM for total (mixed) proteins, the mitochondrial (mito) and sarcoplasmic (sarco) subfractions, and the contractile protein myosin heavy chain (MHC). FR, food restricted; MPred, methylprednisolone. * Less than Control, *P<0.05*, † Higher than FR, *P<0.05*. There was a trend for lower FSR of MHC in plantaris of MPred versus Control, *P* = *0.069*.

Fractional synthesis rates (FSR) are shown in Figure 2. In all groups and in both muscles, the mitochondrial protein FSR was 50–200% higher (*P<0.002*) than the FSR of total mixed, sarcoplasmic or MHC proteins. Within the Control group, mitochondrial, sarcoplasmic and MHC FSR were 39–49% lower in plantaris than soleus (*P<0.015*), though the mixed protein FSR was only 18% lower in plantaris (*P* = *0.15*). In FR group FSR of each protein fraction was 51–69% lower in plantaris than in soleus (*P<0.02*). In the MPred group, sarcoplasmic protein FSR was 41% lower in plantaris (*P<0.002*), while the other fractions were only 15–19% lower (*P*>*0.05*).

Compared to Control values FSR of all soleus protein fractions was reduced by 37–65% (*P<0.015*) in the FR and MPred groups. There were no significant differences in FSR between FR and MPred in the soleus. In the plantaris, the FSR of all protein fractions were reduced by 25–70% in FR and MPred compared to Control. All of these differences reached statistical significance (*P<0.05*) except for MHC in the MPred group, which showed a trend in the same direction (52% lower than Control, *P* = *0.069*). Additionally, FSR of the FR plantaris mixed, mitochondrial, and sarcoplasmic proteins was 19–30% lower (*P<0.02*) compared to MPred.

### Activity of BCAAT and BCKAD (Fig. 3)

BCAAT activity in quadriceps muscle was 85% (*P* = *0.018*) and 79% (*P* = *0.052*) higher in the FR and MPred groups, respectively, compared to Control. BCKAD activity in the same samples was 51% higher in both the FR and MPred groups (*P* = *0.035*) compared to Control.

**Figure 3:**
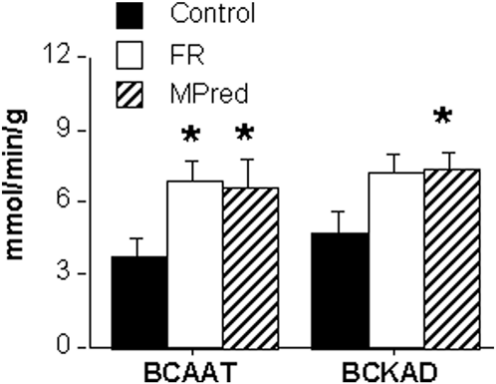
Activity of branched-chain amino acid aminotransferase (BCAAT) and α-keto acid dehydrogenase (BCKAD) in skeletal muscle. Data from red quadriceps muscle (N = 5–8 animals per treatment, mean±SEM). FR, food restricted; MPred, methylprednisolone. * MPred and FR higher than Control, *P<0.05.* There was a trend for higher BCKAD in FR versus Control, *P* = *0.052*.

## Discussion

The main new finding in the current study was that muscle mitochondrial function was largely maintained following 14 d of either methylprednisolone treatment or food restriction, despite the fact that both conditions resulted in loss of muscle mass and reduction in mitochondrial protein synthesis rate. The decline in mixed muscle protein synthesis rate in MPred and FR animals is consistent with prior studies, but we hypothesized that mitochondrial proteins may be differentially regulated from other muscle proteins. A relative preservation of mitochondrial protein synthesis would be an attractive explanation for maintenance of mitochondrial function during catabolic stress but the results show that suppression of protein synthesis affected all protein fractions.

Mitochondrial functional measures in the soleus muscle were not affected by either MPred or FR treatment but the plantaris muscle did show limited changes. FR resulted in a decrease in COX activity in the plantaris muscle but this difference appears to have had minimal impact on ATP generating capacity with either of the substrate combinations used. This suggests that there was either ample reserve capacity of the COX enzyme or other compensatory regulation occurred. In comparison, a reduction in ATP production rate with pyruvate + malate in plantaris muscle was the only mitochondrial function change resulting from MPred treatment. Since ATP production rate with the fatty acid substrate palmitoyl-carnitine was maintained in the MPred plantaris it is likely that an early step specific to pyruvate metabolism, such as transport into the mitochondria, was affected by MPred treatment and responsible for the differential results. This interpretation would also suggest that most of the remaining common pathways leading to ATP synthesis (Kreb's cycle and electron transport chain) remain unchanged during MPred treatment. It is presently unclear why pyruvate metabolism would be selectively affected by glucocorticoid action in the mixed oxidative-glycolytic plantaris but not in soleus., which has higher oxidative capacity. This finding is, however, consistent with the pattern of muscle-specific effects of glucocorticoid action, which has been shown, for example to induce greater atrophy in white/glycolytic muscles compared to the predominantly oxidative soleus [7].

To our knowledge this is the first study to compare mitochondrial ATP production rates in multiple muscles of rats undergoing FR and MPred treatments. The current findings are mostly in agreement with earlier reports showing that short (up to 7 days) or longer term (up to 7 months) food restriction does not impair muscle mitochondrial ATP production or enzymatic activity [14], [16], [17], and may actually lead to increased mitochondrial oxidative capacity in some cases. It was shown, for example, that a 24% reduction in food intake (versus *ad libitum*) for 7 days results in increased mRNA expression of COX enzyme subunits in rat muscle and that activity of COX and citrate synthase are increased by 21 days [16]. It is not clear why those results differed from the present findings, where mitochondrial function and COX mRNA were not increased after 14 days of food restriction. However, the likelihood of mitochondrial enhancement may be greater with longer durations studies as we previously reported that mitochondrial ATP production with palmitoyl-carnitine increased in rat muscle following 7 months of food restriction [17]. As with FR, muscle mitochondrial oxidative capacity is typically preserved in rats treated with glucocorticoids for 10–11 days [4], [6], [35]. For example, despite muscle atrophy there was no change in the activity of citrate synthase or COX, or the abundance of COX subunit mRNAs in plantaris muscle in glucocorticoid-treated rats [4], [6]. There are exceptions, however, as it was reported that COX activity declined in plantaris muscle in dose-dependent fashion in rats treated with corticosterone for 12 days [36]. The opposite effect was shown in study of young rats treated with dexamethasone for 3 days in which cytochrome c content and the abundance of mitochondrial gene transcripts were increased in quadriceps muscle [37]. This latter study differed, however, from most other comparable studies [2], [8], [10], [36], in that a decrease in food intake or body weight was not observed during the 3 day protocol, which may explain those unique findings. The variable responses in mitochondrial function to glucocorticoids may be due to several factors including the form of glucocorticoid administered (which have different potencies [9]), the route of delivery (i.e., daily injections versus continuous pump delivery as used in the present study), the length of treatment, and the strain or age of animals used [2], [36].

We examined the fractional synthesis rate of the total mixed protein pool as well as the mitochondrial, sarcoplasmic and myosin heavy chain subfractions. The finding that synthesis of mitochondrial proteins was consistently higher than other proteins within the same tissue and that synthesis rates of the same proteins varied between tissues (e.g. higher rates in soleus versus plantaris) demonstrates that protein synthesis can be differentially regulated within and between tissues [34], [34,38]. Yet we found that, with limited exception, both MPred and FR treatments resulted in reduced synthesis of all of the protein fractions in both soleus and plantaris muscles when compared to Control, indicating that both FR and MPred treatments have a global suppression effect on muscle protein synthesis rate. Our finding on glucocorticoid effects agrees with a prior report showing that protein synthesis was similarly reduced in glycolytic and oxidative muscles by 5 days of dexamethasone injection [8]. In contrast, another study showed that 6 days of dexamethasone treatment caused a greater decline in mixed muscle protein synthesis rate in gastrocnemius compared to the soleus [9]. To our knowledge the effects of FR on protein synthesis rates in different muscles has not previously been reported.

The decline in muscle protein synthesis in the MPred rats may be partly due to anorexia since similar or greater reductions in protein synthesis were observed in the FR group. Many of the prior studies that reported a reduction in mixed muscle protein synthesis in rats given high doses of glucocorticoids for up to 12 days did not account for the potential effect of reduced food intake [3], [5], [9], [39]. Since energy deficit alone can cause a reduction in muscle protein synthesis rate [12], [13], [40], it is important to control for food intake when these outcomes are analyzed. Among studies implementing pair-feeding, however, the impact of glucocorticoids has not been consistent. In contrast to our results, it was reported that *in vivo* mixed muscle protein synthesis was reduced more in rats injected with dexamethasone for 5 days than in pair-fed controls [8]. In the absence of an ad libitum-fed control group though, it was unclear how much of an effect the reduced food intake *per se* had on protein synthesis. When the same investigators measured protein synthesis in the epitrochlearis muscle using an *in vitro* technique they found no difference between dexamethasone-treated and pair-fed animals [1], [2]. Thus, it is unclear whether the discrepancy among these studies is due to the differences in the muscles tested or the measurement techniques used.

The MPred group had lower body mass and muscle mass than the FR group, demonstrating a greater catabolic effect of glucocorticoids compared to food restriction alone. The difference in muscle mass cannot be explained either by protein synthesis or by muscle hydration, which did not differ among groups, Nor can the differences in muscle loss be attributed to activity of the branched-chain amino acid transamination (BCAAT) and oxidation (BCKAD) enzymes, which were similarly increased in both FR and MPred muscles. Another potential reason for the greater catabolic effect in Mpred group is greater muscle protein catabolism in MPred than FR. This is supported by earlier studies showing that during fasting or energy restriction muscle protein synthesis and breakdown decrease [1], [2], [12], [13], [41], whereas in response to glucocorticoids markers of protein breakdown and proteolytic pathways are elevated [1], [42]. It has been repeatedly shown that glucocorticoid-stimulated muscle protein breakdown is mediated primarily through ubiquitin-proteasome-dependent proteolysis and other calcium-dependent protein degradation pathways [43].

Although measuring breakdown rates of specific tissues or proteins is not yet possible *in vivo*, the tracer data available do provide indirect evidence that protein breakdown was higher in the MPred group versus FR. Since all animals received the same amount of [^13^C_6_] phenylalanine tracer, the higher enrichment in plasma and muscle free pools of the FR group could occur only if the appearance rate of unlabeled phenylalanine from protein breakdown was substantially reduced compared to MPred rats and Controls. There are no alternative explanations that we are aware of to explain these clear differences in plasma and tissue fluid enrichment. Moreover, this interpretation, although relying on an indirect index, can account for the differences in final muscle mass. Thus, while muscle protein synthesis was reduced in the FR group versus Control, the loss of muscle mass was minimized due to a concomitant decline in protein breakdown. The greater loss of muscle mass in the MPred group appears to be due to reduced protein synthesis rate along with protein breakdown that remained similar to the Control group. The failure of protein breakdown to decrease along with protein synthesis would result in negative protein balance and muscle wasting in MPred animals.

Our regimen of methylprednisolone at 4 mg/kg/day for 14 days in young adult rats was selected to ensure that a high pharmacological glucocorticoid effect would be achieved and it is among the longest duration of treatment at a pharmacologic dosage in studies of muscle metabolism. This may account for the strong, sustained decline in food intake and body mass. In comparison, studies in healthy humans have typically been limited to up to one week of moderate-dose treatment, with the effect of increased whole body protein breakdown under post-absorptive conditions in some [44], [45], but not all [46] studies. Under these dose and duration levels, glucocorticoid treatment appears to have no effect on mixed muscle protein synthesis or mitochondrial function in humans [45]–[47] but may increase muscle protein breakdown or net amino acid release (an index of catabolism) in some [45], [47], [48] but not all [46] studies. High dose glucocorticoids and synthetic preparations, such as dexamethasone, are routinely used in acutely ill patients with brain tumors or cerebral edema. To our knowledge though, the impact of these interventions on muscle mitochondrial metabolism and food intake have not been evaluated. Although the effects of glucocorticoids found in our study cannot be directly applied to human subjects receiving moderate doses of glucocorticoids, the effects of energy restriction may be relevant to clinical states such as postoperative recovery when food intake is restricted.

In conclusion, the current study demonstrates that both FR and MPred treatments result in loss of body mass and muscle mass during a 14 d intervention. However, muscle mitochondrial function was largely unchanged in oxidative and mixed oxidative-glycolytic muscles following both FR and MPred. The maintenance of mitochondrial function occurred at the same time there was a ∼40–50% decline in the rate of synthesis of mitochondrial proteins in the same muscles. It is not yet known whether mitochondrial proteins are differentially targeted for breakdown under these conditions or if other compensatory mechanisms may explain the maintenance of mitochondrial function. The finding that the decline in muscle protein synthesis was similar in FR and MPred rats highlights the importance of accounting for changes in food intake in rats receiving glucocorticoids.
